# Quality control and validation of extracellular vesicles isolated from cultured human breast cancer cells

**DOI:** 10.1186/s13104-024-06865-x

**Published:** 2024-07-23

**Authors:** Urvi Patel, David Susman, Alison L. Allan

**Affiliations:** 1 Department of Anatomy & Cell Biology, Western University, London, ON N6A 3K7 Canada; 2 Department of Oncology, Western University, London, ON N6A 5W9 Canada; 3 Verspeeten Family Cancer Centre, London Health Sciences Centre, London, ON N6A 5W9 Canada; 4 London Health Sciences Centre Research Institute, London Health Sciences Centre, London, ON N6A 5W9 Canada

**Keywords:** Extracellular vesicles, Breast cancer, Cell culture, Ultracentrifugation, Immunoblotting, Nanoflow cytometry, Electron microscopy

## Abstract

**Objective:**

Extracellular vesicles (EVs) have been shown to play a critical role in promoting tumorigenesis. As EV research grows, it is of importance to have standardization of isolation, quality control, characterization and validation methods across studies along with reliable references to explore troubleshooting solutions. Therefore, our objective with this Research Note was to isolate EVs from multiple breast cancer cell lines and to describe and perform protocols for validation as outlined by the list of minimal information for studies of EVs (MISEV) from the International Society for Extracellular Vesicles.

**Results:**

To isolate EVs, two techniques were employed: ultracentrifugation and size exclusion chromatography. Ultracentrifugation yielded better recovery of EVs in our hands and was therefore used for further validation. In order to satisfy the MISEV requirements, protein quantification, immunoblotting of positive (CD9, CD63, TSG101) and negative (TGFβ1, β-tubulin) markers, nanoflow cytometry and electron microscopy was performed. With these experiments, we demonstrate that yield of validated EVs varied between different breast cancer cell lines. Protocols were optimized to accommodate for low levels of EVs, and various technical and troubleshooting suggestions are included for potential application to other cell types that may provide benefit to investigators interested in future EV studies.

**Supplementary Information:**

The online version contains supplementary material available at 10.1186/s13104-024-06865-x.

## Introduction

Extracellular vesicles (EVs) are membrane-bound entities that are released into the extracellular space by all cell types [1]. Originally viewed as cellular waste, EVs have now been demonstrated to contain active biomolecules including proteins, lipids and nucleic acids that facilitate intracellular communication [1]. Extracellular vesicles can be divided based on size, function, biogenesis and release pathways [1]. Main subtypes include exosomes (30–150 nm), microvesicles (100–1000 nm), and apoptotic bodies (50–5000 nm) [1, 2]. There is a growing body of research demonstrating the involvement of EVs in cancer. Reciprocal relationships exist whereby changes to the microenvironment induced by tumorigenesis also influence the production and packaging of EVs [3]. In particular, breast cancer cells produce EVs that enhance pro-tumorigenic traits such as growth, migration, angiogenesis, pre-metastatic niche formation and metastasis [4–8]. As understanding of EVs and their role in cancer grows, studies have also explored their use as potential biomarkers and/or therapeutic carriers to deliver anti-cancer drugs [9–12].

With the EV field rapidly expanding, it is important that isolation and analysis methods are standardized. This has been a challenging goal due to the heterogenous nature of EVs and the various biological sources they can be derived from, including cell culture media, plasma, urine and other bodily fluids, and solid tissue [13]. To provide standardization parameters for the EV field, the International Society for Extracellular Vesicles has published guidelines related to minimal information for studies of extracellular vesicles (MISEV) which encompass experimental and reporting requirements to consider when conducting EV research [14–17]. These guidelines are designed to increase rigor and reproducibility of EV studies and to standardize reporting of experimental parameters for studies using EVs from multiple biological sources [14–17]. As per current MISEV guidelines, most separation techniques cannot specifically capture and/or characterize different subtypes of EVs, therefore the term EVs is preferred over biogenesis-based terms and will be used throughout this Research Note [14]. Due to our interest in breast cancer biology and EVs [18, 19], our primary objective was to isolate EVs collected from media from cultured human breast cancer cell lines and to perform the necessary quality control, characterization and validation studies outlined by the 2023 MISEV guidelines [14]. We present an example of how to isolate and validate EVs from cell culture media and include troubleshooting recommendations to overcome common experimental obstacles. Through this process, we gained insight on EV production by breast cancer cells in vitro and optimized protocols to characterize isolated EVs.

## Methods

### Cell Culture

Immortalized human breast-derived cell lines MCF10A, SUM159, MDA-MB-231, MDA-MB-231-4175-LM2 (231-LM2), and MDA-MB-231-1833-BoM (231-BoM) were cultured under hypoxic conditions. Detailed culture conditions described in Additional File 1.

### EV isolation

Cell lines were grown in complete growth media to ~ 80% confluency in 150 mm dishes. Media was changed to serum-free and cells were incubated under hypoxia (~ 1–2% O_2_) for 48 h to optimize EV production and packaging [20]. Higher starting confluencies of 85–90% were required for slow-growing cell lines such as MCF10A to reach appropriate harvesting confluency (95%) after hypoxia treatment. Media was harvested and EVs were isolated using either ultracentrifugation or size exclusion chromatography (SEC) as described in Additional File 1. If EV isolation was not performed immediately after harvesting, culture media was stored at -80 °C.

### Protein isolation, quantification, and immunoblotting

Isolated EVs were lysed using 0.1% NP-40 + protease inhibitor. Total protein concentration in EV samples was quantified using the DC protein assay kit (BioRad). For low-yield EV samples, quantification was completed using the Micro BCA protein assay kit (ThermoFisher Scientific), with samples diluted 10-fold to meet assay volume requirements. A consistent volume (35µL) of each EV-derived protein sample was subjected to immunoblotting as described in Additional File 1.

### Nanoflow Cytometry

Samples of EVs were diluted 10-fold and duplicates samples were analyzed on an Apogee A60 Microplus Nanoflow Cytometer (Apogee Flow Systems Inc., Northwood UK). Specific analysis parameters are detailed in Additional File 1.

## Transmission Electron Microscopy (TEM)

All TEM was carried out at the Canadian Centre for Electron Microscopy (McMaster University, Hamilton, Canada). Detailed TEM methods are provided in Additional File 1.

## Results and discussion

### EV isolation

**Fig. 1:**
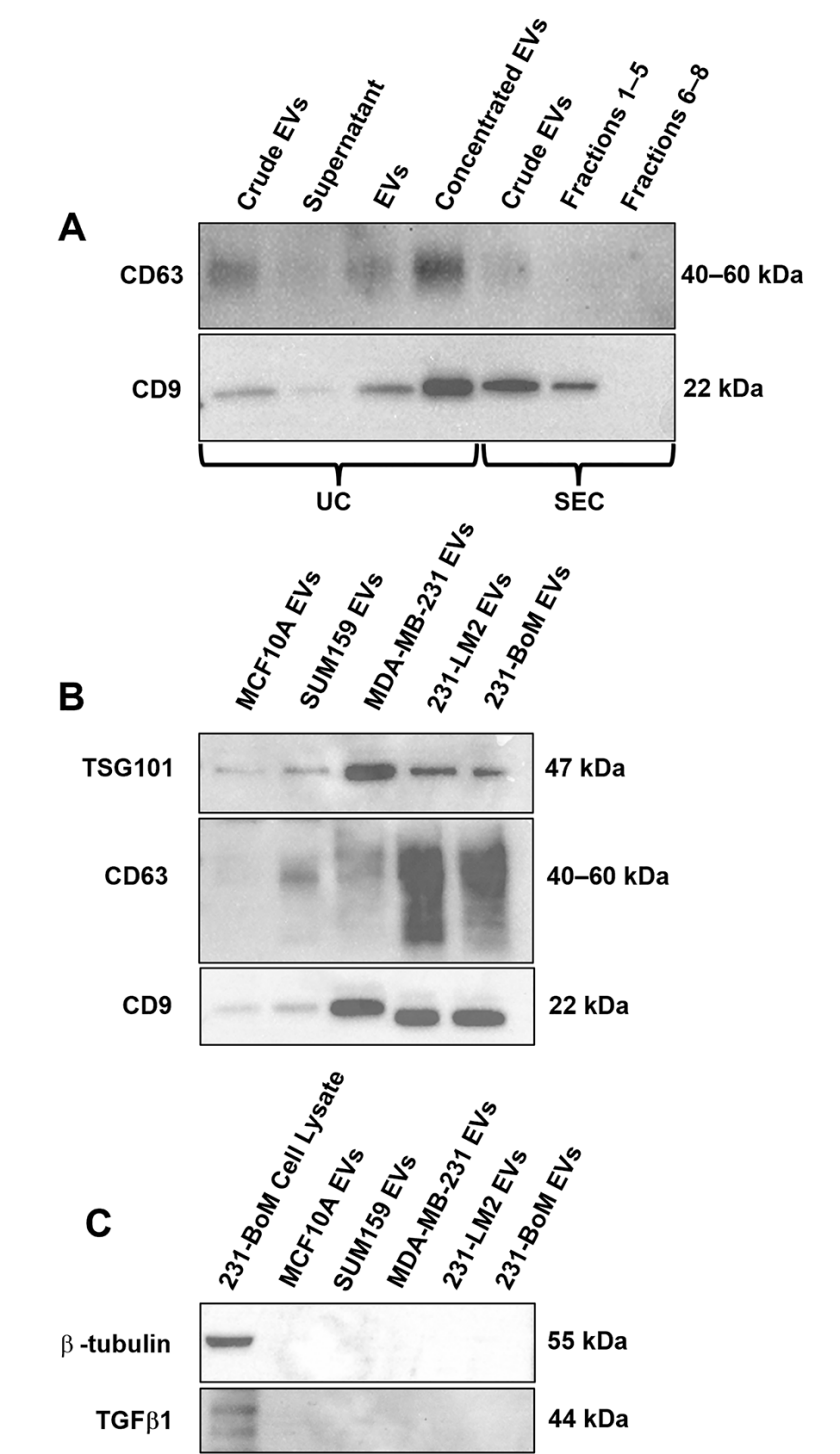
(***A***) Protein levels of CD9 and CD63 following EV isolation by ultracentrifugation (UC) or size exclusion chromatography (SEC). SUM159 cells were cultured under hypoxic conditions and EVs were isolated from cell culture media using UC or SEC as described in the Methods section and in Additional File 1. A consistent volume (35µL) of each EV-derived protein sample was subjected to immunoblotting. Lanes 1–4 show EVs isolated by UC: Lane 1 = crude EVs (media) before UC; Lane 2 = supernatant fraction after UC; Lane 3 = EV fraction after UC; Lane 4 = EV fraction after additional concentrating using an Ultra-4 Centrifugal Filter (100 kDa). Lanes 5–7 show EVs isolated by SEC: Lane 5 = crude EVs (media); Lane 6 = EVs from Fractions 1–5 of the SEC (pooled); Lane 7 = EVs from Fractions 6–8 of the SEC (pooled). (***B***,*** C***) To characterize EVs by protein composition, ultracentrifugation was used to isolate EVs from different breast cell lines cultured under hypoxic conditions including MCF10A (non-malignant breast epithelial), SUM159, MDA-MB-231 (triple negative), 231-BoM (bone metastasizing triple negative), and 231-LM2 (lung metastasizing triple negative). Immunoblots showing ***(B)*** Category 1 transmembrane markers CD9 and CD63, and Category 2 cytosolic marker TSG101; and ***(C)*** Category 3 purity control marker TGFβ1 plus negative protein marker β-tubulin. Cell lysates from 231-BoM cells were used as a positive control for TGFβ1 and β-tubulin. Uncropped blots are presented in Additional File 3

We first carried out a preliminary assessment comparing 2 different methods for EV isolation using human SUM159 breast cancer cells cultured under hypoxic conditions. This included ultracentrifugation and SEC followed by immunoblotting for the transmembrane EV markers CD9 and CD63 [21]. Based on CD9 and CD63 detection, crude EVs (unpurified) were present and detectable in culture media prior to ultracentrifugation or SEC (Fig. 1A). After ultracentrifugation, both markers were mainly observed in the EV fraction, with only a faint band in the supernatant, indicating successful isolation. The EV fraction was further concentrated using an Ultra-4 Centrifugal Filter (100 kDa), resulting in increased marker expression. In contrast, there was a reduction in CD9 and no presence of CD63 after SEC isolation (fractions 1–5) relative to crude EV samples (Fig. 1A). No presence of CD9 and CD63 was observed in fractions 6–8; an observation that was expected as these fractions are outside the ideal collection volume. Overall, in our hands, EVs isolated by SEC demonstrated lower CD9 and CD63 expression than ultracentrifugation in this preliminary assessment, and thus we chose to focus all further validation experiments on characterizing EVs that were isolated only by ultracentrifugation.

### EV quantification

Five different human breast-derived immortalized cell lines cultured under hypoxic conditions were used including one non-malignant breast epithelial line (MCF10A) and four breast cancer lines (SUM159, MDA-MB-231, 231-LM2, 231-BoM). The MISEV guidelines indicate that EVs should be defined quantitatively with regards to their source, such as indicating the number of secreting cells [14]. Accordingly, at harvest, we performed cell counts which typically fell within the range of 1.5–2.2 × 10^7^ cells for each 150 mm dish. The MISEV guidelines also suggest that approximate abundance of EVs can be assessed through protein quantification [14]. Thus, EV samples were quantified using a DC protein assay. In some cases, EV concentrations were consistently low, especially from SUM159 cells, resulting in low absorbance values and negative concentrations (Additional File 2 A). Therefore, low-yield EV samples were quantified using a Micro BCA assay which was able to appropriately quantify lower protein concentrations (Additional File 2B).

### Characterization of EVs by protein composition

For EV characterization, MISEV guidelines recommend using orthogonal methods since a single measurement is not able to fulfil all EV characterization requirements [14]. The first recommended method used was characterizing isolated EVs based on protein composition. The MISEV guidelines list 5 categories for protein-based characterization, recommending that at least one protein each from Categories 1 and 2 (confirming presence of EV features) and at least 1 protein from Category 3 (confirming purity from common contaminants) be assessed [14]. Using immunoblotting, we observed the presence of 3 positive EV protein markers including 2 transmembrane markers (CD9, CD63; Category 1) and 1 cytosolic marker (TSG101; Category 2) (Fig. 1B). For Category 3, we assessed for the absence of TGFβ1 and a second negative control marker, β-tubulin [14]. As expected, both markers were present in breast cancer cell lysates but not in EV samples (Fig. 1C). Due to variation in EV yield (and thus protein abundance) between different cell line sources, samples were loaded at a set volume rather than a set concentration for these validation studies. As a result, for low yield EV samples such as MCF10A and SUM159, EV markers were consistently present at a lower abundance than in higher yield cell lines such as MDA-MB-231 (Fig. 1B, C). To address this in the future, samples should be loaded based on protein concentration. In addition, imaging agents with low femtogram-level sensitivity could be used to detect low abundance proteins [22, 23].

### Particle analysis characterization of EVs

**Fig. 2:**
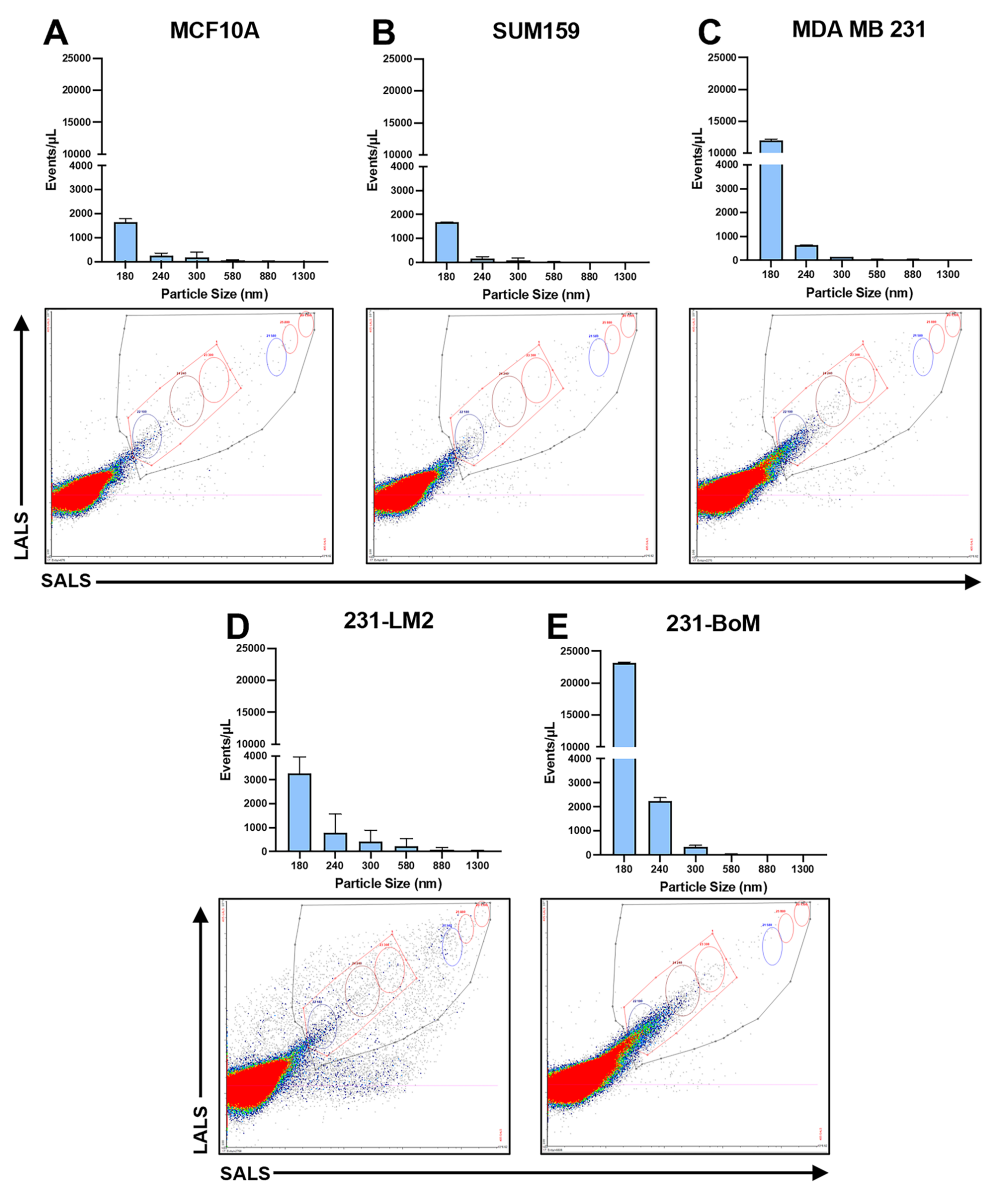
Characterization of particle size distribution profiles of breast-derived EVs. Ultracentrifugation was used to isolate EVs from breast cell lines cultured under hypoxic conditions including MCF10A, SUM159, MDA-MB-231, 231-BoM, and 231-LM2. Each EV sample was diluted 10-fold in PBS and 300µL of the sample was subjected to nanoflow cytometry using an Apogee A60 Microplus Nanoflow Cytometer. ***(A-D)*** Particle number (*y-axis*) and particle size (*x-axis*) distribution in EV samples from ***(A)*** MCF10A, ***(B)*** SUM159, ***(C)*** MDA MB 231, *(****D)*** 231-LM2, and ***(E)*** 231-BoM breast cell lines. Events/µL shown as mean ± standard deviation (*n* = 2) and normalized to cell number at time of harvest. Cytograms show small angle light scatter (SALS) versus large angle light scatter (LALS) with quantification depicted in the graphs. Standardized beads of varying sizes (180 nm, 240 nm, 300 nm, 580 nm, 880 nm and 1300 nm) are depicted as circles on the cytogram

Nanoflow cytometry was also carried out to assess particle size distribution profile and relative abundance of particles within EV samples (Fig. 2). For all breast-derived EV samples obtained under hypoxic conditions, the majority of EVs fell within the size category of 180 nm, although there were also EVs of larger sizes detected in specific cell lines. In keeping with this and similar to the immunoblotting results, we also observed variability in EV yield (events/µL) between cell lines, with MCF10A and SUM159 cells having the lowest EV yield (Fig. 2A, B) and 231-BoM cells having the highest EV yield (Fig. 2E). This variability in both EV size and yield illustrates the inherent heterogeneity between breast cancer cells isolated from different patients and underscores the importance of using multiple cell line models for cancer EV studies.

### Characterization of EV morphology

**Fig. 3:**
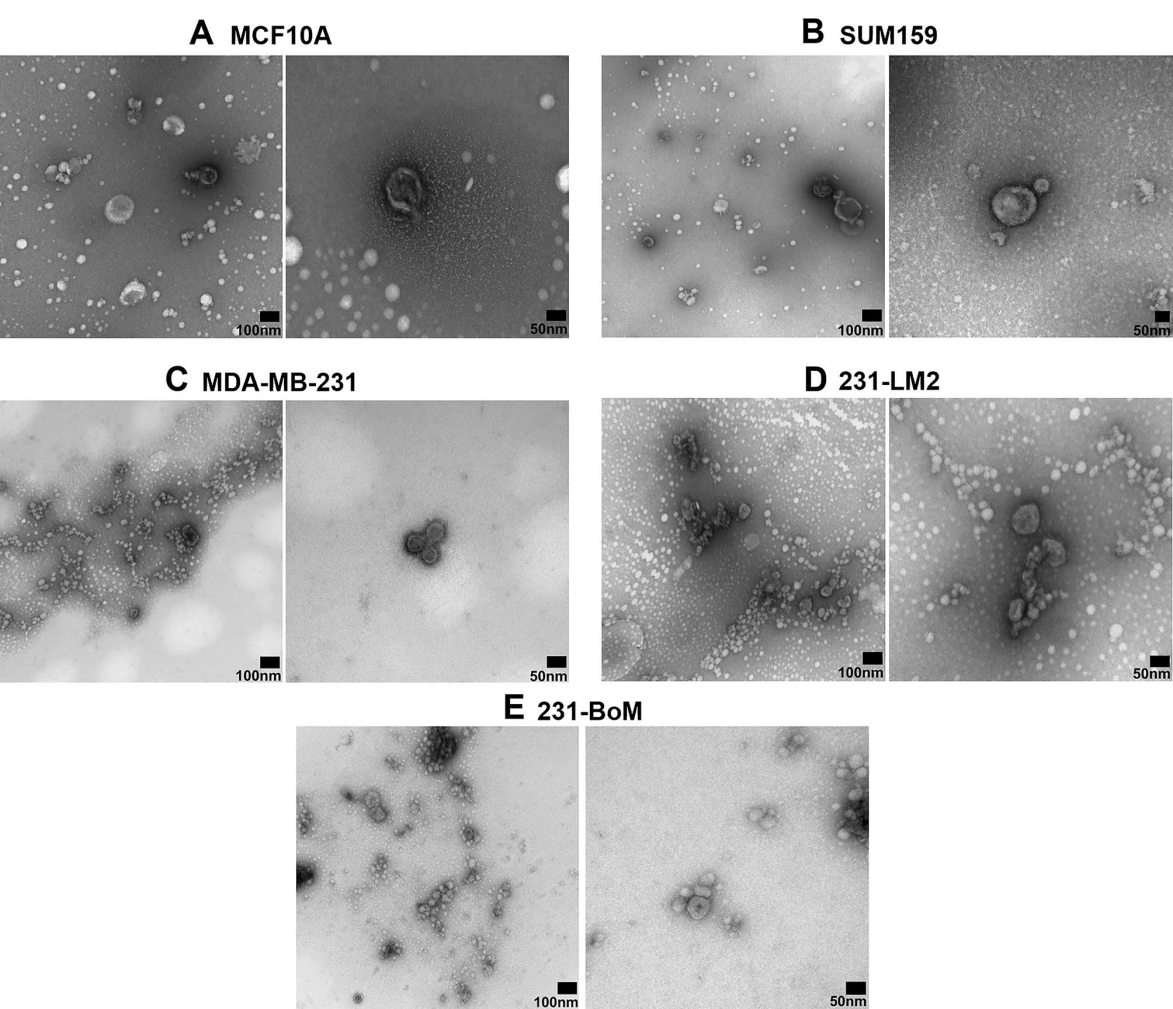
Characterization of EV morphology by transmission electron microscopy (TEM). Ultracentrifugation was used to isolate EVs from different breast cell lines cultured under hypoxic conditions including MCF10A, SUM159, MDA-MB-231, 231-BoM, and 231-LM2. Samples were prepared in 3 µL volumes in PBS and subjected to TEM using a JEOL JEM 1200 EX TEMSCAN microscope. **(A)** MCF10A, **(B)** SUM159, **(C)** MDA MB 231, **(D)** 231-BoM, and (**E)** 231-LM2. Images were captured at an accelerating voltage of 80 kV at magnifications of 100,000x (*wide-field*,* left panels*) or 200,000x (*close-up*,* right panels*) with the exception of SUM159 EVs where close-up images were captured at 150,000x

Finally, MISEV guidelines recommend using high-resolution imaging techniques such as TEM to characterize EV morphology [14]. We performed TEM with negative staining and, consistent with previous literature [24], observed that breast-derived EVs isolated under hypoxic conditions could be visualized as round-shaped structures (Fig. 3). However, depending on the preparation and reagents, EVs can also appear cup-shaped [24]. With high-resolution TEM, there may be background contaminants such as cell debris which can interfere with EV imaging. By introducing additional PBS washes, ensuring pellets are thoroughly resuspended, and rinsing the grid to remove buffer salts, image quality can be improved. Removing contaminants also allows for imaging without dilution which is crucial for low yield samples (i.e., from MCF10A, SUM159 cells) in which EVs are more difficult to detect.

## Conclusions

The validation experiments described here allow for sufficient EV characterization to satisfy MISEV recommendations and provide quality-assurance of EVs. These experimental parameters can also be reported in EV-TRACK [25, 26] to improve reproducibility of EV research, and validated EVs can subsequently be used for functional experiments. For example, in the cancer biology setting EVs can be used in vitro to assess their influence on cell behaviour, or in vivo to observe effects on the tumor microenvironment and/or disease progression. In addition, detailed characterization of molecular cargo contained within EVs can be profiled to discover biomarkers or be applied for therapeutic purposes such as drug delivery.

In summary, when conducting an EV study, it is important to consider the following factors: terminology and definitions being used; source of EVs; and techniques used for separation, concentration, characterization, and storage. Documenting these parameters will assist in satisfying MISEV guidelines and ensure that methods are reported to allow for replication. The validation protocols described in this Research Note will be valuable to investigators interested in studying EVs, including as a reference to overcome common issues encountered during EV studies and to help fulfil MISEV requirements that ensure standardization across the EV field.

### Limitations

The main limitation in isolation/characterization of EVs results from varying levels of EV yield across different cell types, or even within particular cell types with different origins. Yield from certain breast cancer cell lines were low which produced challenges during isolation, validation and subsequent functional studies. The best example of this was SUM159 cells, which we observed to consistently have the lowest EV yield of all the human breast-derived cell lines examined. To manage this limitation experimentally during isolation by ultracentrifugation, investigators should be aware that the EV pellet is not always visible to the naked eye and thus requires extra care during washes and resuspension to avoid EV loss and ensure the greatest EV recovery. In addition, pooling together media from multiple plates and resuspending EVs in low volumes of PBS is recommended to further ensure optimal EV recovery, particularly for low-yield cell models. Another limitation of this study is that we only used one culture condition (hypoxia) during EV isolation as it mimics the tumor microenvironment and enhances EV production [20]. However, depending on the nature of the study, other factors can be introduced to stimulate EV production such as three-dimensional culture conditions, physical or chemical stimulation of cells, and/or genetic manipulation [27]. Furthermore, due to the size detection limits of nanoflow cytometry, smaller sizes of EVs (< 180 nm) cannot be detected using this technology, although some of these smaller EVs can be identified through the complementary TEM analysis. In addition, while we chose to use ultracentrifugation as our isolation procedure, it has been shown that this technique may produce lower purity EV samples compared to Sect. [28] This is highlighted in the TEM analysis, where dark clouds around EVs indicating some protein aggregation. Finally, it has been observed that multiple freeze/thaw cycles can result in loss of EVs [29]. Long term storage at -80 °C can result in degradation of EVs over time [29] and samples may require re-quantification prior to use, so ideally EV samples should be used immediately after isolation, or aliquoted into smaller volumes for future use.

### Electronic supplementary material

Below is the link to the electronic supplementary material.

Supplementary Material 1

Supplementary Material 2

Supplementary Material 3

## Data Availability

No datasets were generated or analysed during the current study.

## Abbreviations

EV: Extracellular vesicle
MISEV: Minimal information for studies of extracellular vesicles
ATCC: American Type Culture Collection
DMEM: Dulbecco’s Modified Eagle’s Medium
FBS: Fetal bovine serum
TN: Triple negative
HEPES: 4-(2-hydroxyethyl)-1-piperazineethanesulfonic acid
SEC: Size exclusion chromatography
PBS: Phosphate-buffered saline
DC: Detergent compatible
BCA: Bicinchoninic acid
SDS-PAGE: Sodium dodecyl sulfate polyacrylamide gel electrophoresis
PVDF: Polyvinylidene difluoride
SM: Skim milk
TBS-T: Tris buffered saline + 0.1% Tween 20
BSA: Bovine serum albumin
CD: Cluster of differentiation
TSG101: Tumor susceptibility gene 101
TGFβ1: Transforming growth factor β1
IgG: Immunoglobulin G
ECL: Enhanced chemiluminescence
SALS: Small angle light scatter
LALS: Long angle light scatter
TEM: Transmission electron microscopy
EV-TRACK: Transparent reporting and centralizing knowledge in extracellular vesicle research
