# Supplementary material for: Quality control and validation of extracellular vesicles isolated from cultured human breast cancer cells
Source: BMC Res Notes. 2024 Jul 23;17:202. doi: 10.1186/s13104-024-06865-x (PMC11265473; doi:10.1186/s13104-024-06865-x)
Supplement: Supplementary file 1 — Supplementary Material 1 [file 13104_2024_6865_MOESM1_ESM.docx]

***Additional File 1***

**Detailed Methods**

**Cell Culture**

Immortalized human breast-derived cell lines MCF10A (non-malignant breast epithelial), SUM159, MDA-MB-231 (triple-negative), MDA-MB-231-4175-LM2 (231-LM2; triple-negative lung-metastasizing), and MDA-MB-231-1833-BoM (231-BoM; triple-negative bone-metastasizing) were used in this study. The MCF10A cell line was obtained from American Type Culture Collection (ATCC; Manassas, VA, USA) and cultured in Dulbecco’s Modified Eagle’s Medium/Nutrient Mixture F-12 (DMEM/F-12) supplemented with 5% fetal bovine serum (FBS), 1% insulin, 0.08% epidermal growth factor, 0.05% hydrocortisone, 0.005% cholera toxin. The human MDA-MB-231 triple negative (TN) cell line was obtained from ATCC and cultured in DMEM/F-12 supplemented with 10% FBS. The 231-LM2 TN lung-metastasizing cell line (20) and the 231-BoM TN bone-metastasizing cell line (21) were a kind gift from Dr. Joan Massagué (Memorial Sloan Kettering Cancer Centre, New York, NY) and were cultured in DMEM supplemented with 10% FBS. The human SUM159 TN cell line was obtained from Asterand Inc. (Detroit, MI) and cultured in Ham’s F-12 supplemented with 5% FBS, 0.5% insulin, 0.1% hydrocortisone, 1% HEPES. Cell lines were authenticated through third party testing in January 2024 (RADIL/IDEXX BioAnalytics, Columbia, MO). All media/supplements were from ThermoFisher Scientific (Carlsbad, CA) and FBS was from Wisent Bioproducts Inc (Saint-Jean-Baptiste, QC, Canada).

**EV Isolation**

Harvested cell culture media was centrifuged for 10 minutes at 1000g to remove cells and debris. Supernatants were concentrated using EMD Amicon Ultra-15 Centrifugal Filters (100 kDa; Millipore Sigma) via centrifugation for 5 minutes at 3500g.

*Ultracentrifugation*

Filtrates were centrifuged using a Sorvall Discovery 90 SE Ultracentrifuge for 1 hour at 100,000g in a swinging bucket rotor (TH-641) with acceleration and deceleration set to mode 9. Polyallomer thin wall tubes (Thermo Scientific, #03-699) were filled with ~11.5 mL of filtrate or topped off with Dulbecco’s phosphate-buffered saline (PBS) to meet the volume requirement to prevent tubes from collapsing. Following ultracentrifugation, supernatants were carefully discarded, EV pellets were washed once and resuspended in Dulbecco’s PBS, concentrated using an Ultra-4 Centrifugal Filter (100kDa; Amicon) and filtered through 0.22 μm filters (Millipore; Billerica, MA). All ultracentrifugation and centrifugation steps were carried out at 4°C and resulting isolated EV samples were stored at -80°C prior to subsequent use.

*Size Exclusion Chromatography (SEC)*

Filtrates were passed through a qEVoriginal Gen 2 35nm column from Izon (Christchurch, New Zealand) using the automatic fraction collector following manufacturer’s protocol. The qEVoriginal column contained an agarose resin matrix, pore size of 35nm, height of 79mm, and diameter of 15.65mm. The void volume was 2.5mL, fraction volume was 0.4mL and fractions 1-8 were collected. The buffer used was Dulbecco’s PBS. Collected fractions were pooled together (Pool 1: fractions 1-5 and Pool 2: fractions 6-8), concentrated using EMD Amicon Ultra-4 Centrifugal Filters 100kDa (Millipore Sigma) via centrifugation for 5 min at 3500g, and filtered through 0.22 μm filters (Millipore; Billerica, MA). All centrifugation steps were carried out at 4°C and resulting isolated EV samples were stored at -80°C prior to subsequent use.

**Immunoblotting**

Proteins were isolated from EV samples and separated using sodium dodecyl sulfate polyacrylamide gel electrophoresis (SDS-PAGE) and transferred onto a polyvinylidene difluoride (PVDF) membranes. Membranes were blocked using 5% skim milk (SM) in tris-buffered saline with 0.1% Tween-20 (TBST) or 5% bovine serum albumin (BSA) in TBST for 1 hour, with the exception of TGFβ1 which was blocked overnight. Primary antibodies including CD9 (Cell Signaling; #13174), CD63 (Abcam, #ab134045), TSG101 (Abcam, #ab83), TGFβ1 (Invitrogen, #MA5-15065) and β-tubulin (Cell Signaling; #2146) were used for immunoblotting using the conditions detailed in *Table 1*.

**Table 1. Primary Antibodies and conditions used for immunoblotting.**

| **Antigen** | **Clone** | **Dilution** | **Diluent** | **Incubation Time** |
| --- | --- | --- | --- | --- |
| Anti-human CD9 | D8O1A | 1:1000 | 5% SM | O/N |
| Anti-human CD63 | EPR5702 | 1:1000 | 5% BSA | 2 h |
| Anti-human TSG101 | 4A10 | 1:1000 | 5% SM | O/N |
| Anti-human TGFβ1 | F.888.7 | 1:1000 | 5% BSA | 2 h |
| Anti-human β-tubulin | 2146 | 1:1000 | 5% BSA | O/N |

SM: Skim milk; BSA: Bovine serum albumin; O/N: Overnight

Goat anti-rabbit IgG and goat anti-mouse IgG secondary antibodies (Millipore Sigma) conjugated to horseradish peroxidase were diluted 1:2000 in TBST+5% SM or TBST+5% BSA. Membranes were incubated with secondary antibodies for 1 hour and protein expression was visualized using Amersham ECL Prime Detection Reagent (GE Healthcare, Wauwatosa, WI).

**Nanoflow Cytometry**

Before running EV samples on the Apogee A60 Microplus Nanoflow Cytometer (Apogee Flow Systems Inc., Northwood UK), a negative control sample of PBS alone was analyzed to verify that background detection levels of particles were below 100 events/s. Standardized beads of varying sizes (180nm, 240nm, 300nm, 580nm, 880nm and 1300nm) (Apogee, #1493) were analyzed as size standards/positive controls and confirmed to be at their stock concentration of 5000 events/s. Data is presented as events/μL (mean ± standard deviation) (n=2) normalized to cell number at time of harvest. To ensure that samples were homogenous, it was ensured that the coefficient of variance was under 25. Higher values indicate heterogenous samples that may result in uneven sample flow rate and produce skewed data. Samples were analyzed at a flow rate of 1.50 μL/min and sheath pressure of 150 mbar. Light scatter of events was done using the 405nm laser with thresholds of 37 a.u. for small angle light scatter (SALS) and 30 a.u. for long angle light scatter (LALS) to remove background noise. The photomultiplier tube voltages were: SALS (340 V), LALS (260 V). EVs were analyzed using the Apogee Histogram Software (Apogee Flow Systems Inc) and graphs were created using GraphPad Prism 10 (GraphPad Software, Boston, MA, USA).

**Transmission Electron Microscopy (TEM)**

Extracellular vesicle (EV) samples were prepared as described above from different breast cell lines including MCF10A, SUM159, MDA-MB-231, 231-BoM, and 231-LM2. For each EV sample, 3 μL was pipetted onto a Formvar coated 200 mesh Cu grid and incubated for 10 minutes. Excess liquid was removed with filter paper by blotting from the sides. The grid was washed twice by brief contact with MilliQ water droplets and excess liquid was removed with filter paper by blotting from the side. For 1 minute, 3 μL of 1% aqueous uranyl acetate (Canemco Inc., QC) was added to the grid and then blotted from the side with filter paper. A JEOL JEM 1200 EX TEMSCAN microscope (JEOL, Peabody, MA, USA) was used to view the grids at an accelerating voltage of 80kV. Images were captured with an AMT 4-megapixel digital camera (Advanced Microscopy Techniques, Woburn, MA) at magnifications of 100,000x (*wide-field, left panels*) and 200,000x (*close-up, right panels*), with the exception of SUM159 EVs, for which close-up images were captured at 150,000x.
