# Supplementary figures and images for: Quality control and validation of extracellular vesicles isolated from cultured human breast cancer cells
Source: BMC Res Notes. 2024 Jul 23;17:202. doi: 10.1186/s13104-024-06865-x (PMC11265473; doi:10.1186/s13104-024-06865-x)

Additional File 2

A

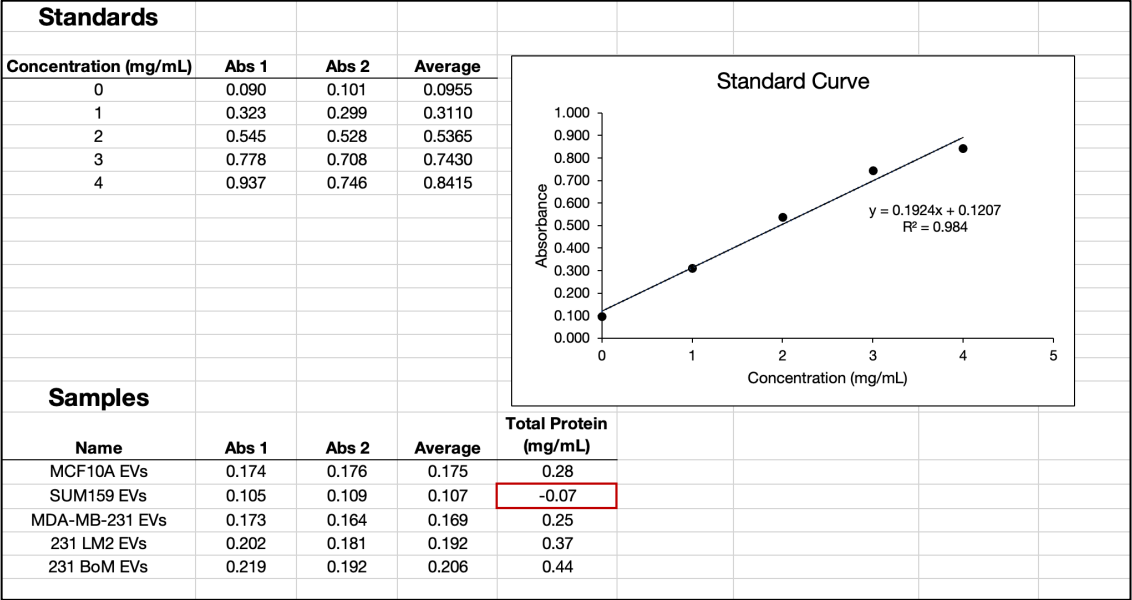

B

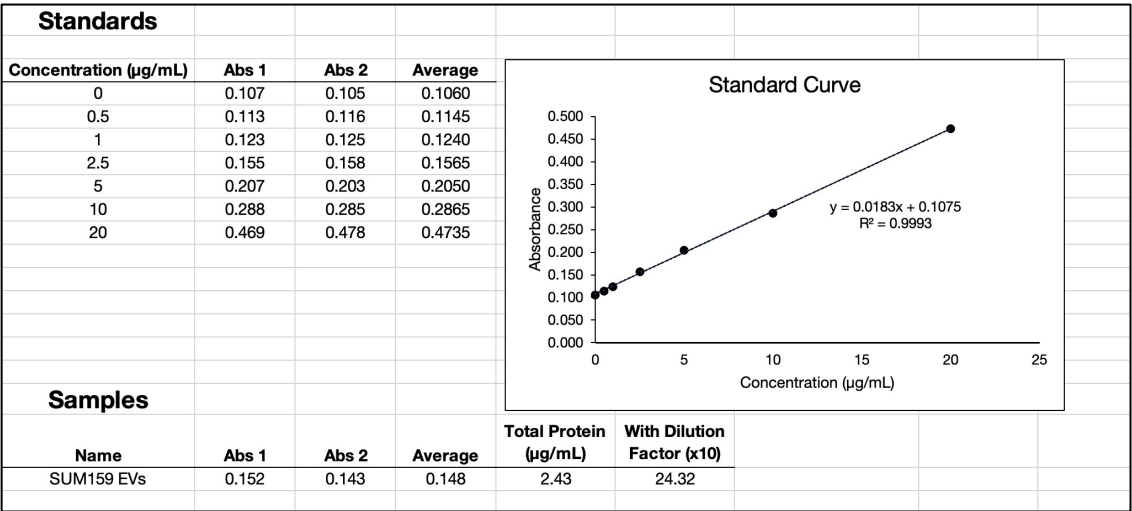

Supplement: Supplementary file 2 — Supplementary Material 2 [file 13104_2024_6865_MOESM2_ESM.pdf]

### Additional File 3

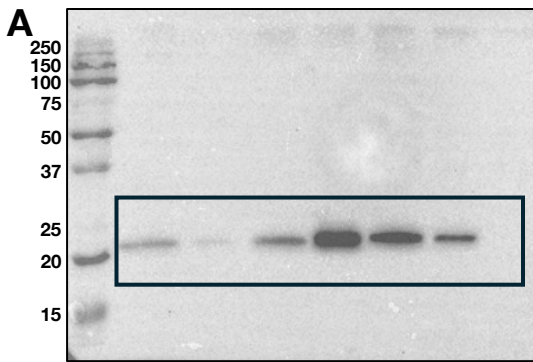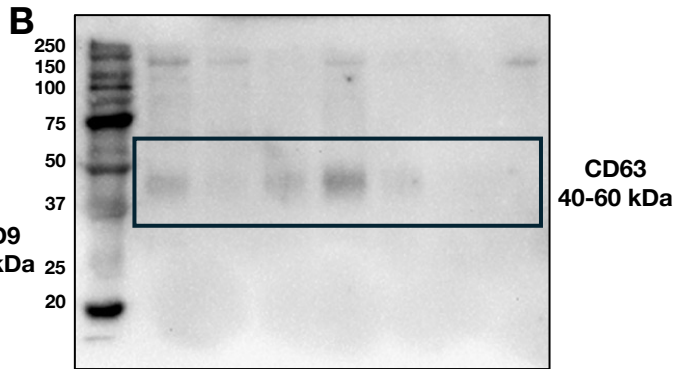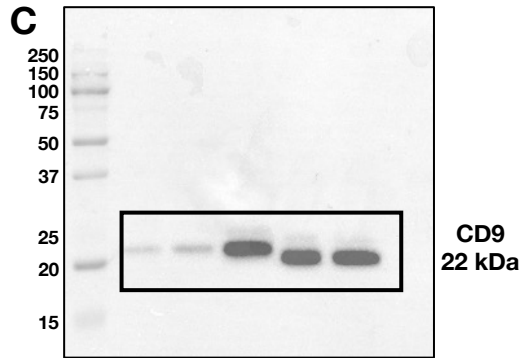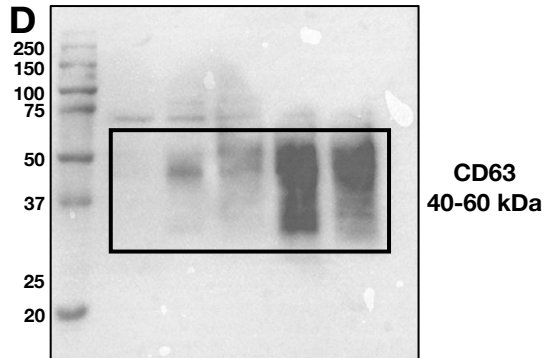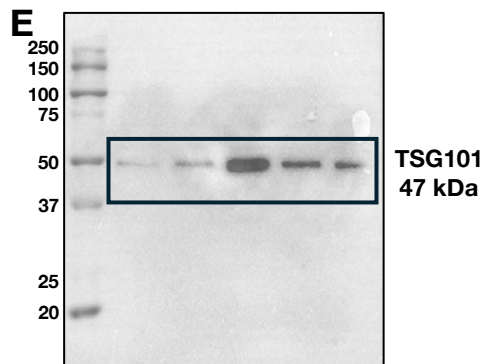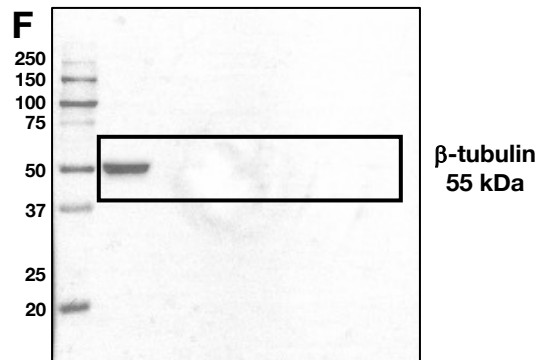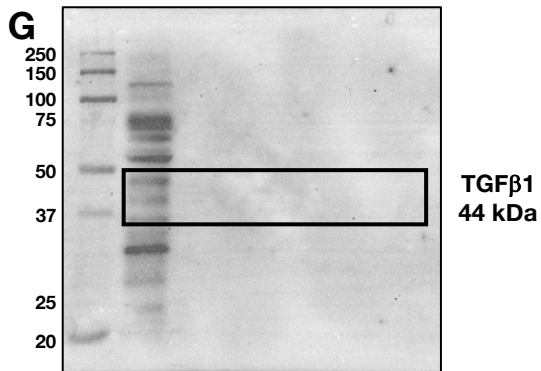

Supplement: Supplementary file 3 — Supplementary Material 3 [file 13104_2024_6865_MOESM3_ESM.pdf]
